# Improved multi-parametric prediction of tissue outcome in acute ischemic stroke patients using spatial features

**DOI:** 10.1371/journal.pone.0228113

**Published:** 2020-01-24

**Authors:** Malte Grosser, Susanne Gellißen, Patrick Borchert, Jan Sedlacik, Jawed Nawabi, Jens Fiehler, Nils Daniel Forkert

**Affiliations:** 1 Department of Diagnostic and Interventional Neuroradiology, University Medical Center Hamburg-Eppendorf, Germany; 2 Department of Radiology and Hotchkiss Brain Institute, University of Calgary, Calgary, Canada; University Hospital Basel, SWITZERLAND

## Abstract

**Introduction:**

In recent years, numerous methods have been proposed to predict tissue outcome in acute stroke patients using machine learning methods incorporating multiparametric imaging data. Most methods include diffusion and perfusion parameters as image-based parameters but do not include any spatial information although these parameters are spatially dependent, *e*.*g*. different perfusion properties in white and gray brain matter. This study aims to investigate if including spatial features improves the accuracy of multi-parametric tissue outcome prediction.

**Materials and methods:**

Acute and follow-up multi-center MRI datasets of 99 patients were available for this study. Logistic regression, random forest, and XGBoost machine learning models were trained and tested using acute MR diffusion and perfusion features and known follow-up lesions. Different combinations of atlas coordinates and lesion probability maps were included as spatial information. The stroke lesion predictions were compared to the true tissue outcomes using the area under the receiver operating characteristic curve (ROC AUC) and the Dice metric.

**Results:**

The statistical analysis revealed that including spatial features significantly improves the tissue outcome prediction. Overall, the XGBoost and random forest models performed best in every setting and achieved state-of-the-art results regarding both metrics with similar improvements achieved including Montreal Neurological Institute (MNI) reference space coordinates or voxel-wise lesion probabilities.

**Conclusion:**

Spatial features should be integrated to improve lesion outcome prediction using machine learning models.

## Introduction

Acute ischemic stroke is one of the major causes of mortality and disability [1]. At the same time, various socioeconomic factors are also related to this disease, *e*.*g*. reduced quality of life of the patient and families, emotional distress, and high costs for the health care system. An ischemic stroke originates from an occlusion of an artery supplying the brain with blood, which results in a shortage of oxygen, glucose, and supply of other essential nutrients, ultimately leading to a necrosis. An occlusion does not lead to an immediate necrosis of the whole affected brain tissue due to blood flow via collateral connections. However, the ability of brain tissue to endure this hypoperfusion is limited leading to a dynamic growth of the necrosis until the whole brain tissue mainly supplied by the occluded artery is necrotic.

Imaging parameters derived from diffusion- and perfusion-weighted MR imaging (DWI and PWI) are known to correlate with the voxel-wise infarct outcome [2, 3]. Owing to the high complexity of cerebral perfusion and metabolism and dynamic lesion growth, thresholding a single parameter map from PWI and DWI, as commonly done in the clinical routine for tissue outcome prediction, oversimplifies the reality [4]. Therefore, a good predictive model should aim to include as much of the available imaging data as possible. Considering this, several multi-parametric tissue outcome prediction methods have been proposed in the past utilizing multiple imaging parameters to predict tissue outcome after an acute ischemic stroke [3, 5, 6]. These methods utilize various machine learning approaches such as tree ensembles and artificial neural networks [7–9]. Generally, incorporating multi-parametric image information has been shown to lead to better outcome predictions compared to single parameter thresholding. However, most previously published multi-parametric outcome prediction methods do not include any spatial information, essentially treating all voxels in the same way although it is known that even white and gray brain matter have considerably different perfusion properties [10]. However, not only white and gray matter brain tissue exhibit different sensitivity to endure hypoperfusion, but even different anatomical and functional brain regions might differ in this respect. Thus, the anatomical location beyond simple white and gray matter differentiation in the brain might play a crucial role for predicting the tissue outcome with high accuracy.

Thus, the aim of this study was to investigate if integrating spatial information into the machine learning model can improve the accuracy of multi-parametric tissue outcome prediction.

## Materials and methods

### Patients

We retrospectively analyzed data of patients with anterior circulation strokes collected from 2006 to 2009 in five different centers. The study was approved by the local ethics committees and institutional review boards (University Centre Hamburg-Eppendorf, Germany).

If possible, patients were treated with intravenous tissue-type plasminogen activator (IV tPA). Criteria for patient inclusion in this study included: (1) first-ever stroke, (2) National Institutes of Health Stroke Scale (NIHSS) > 4, (3) multiparametric MRI, including PWI and DWI ≤ 12 hours of witnessed stroke onset, (4) follow-up MRI, including a FLAIR MRI dataset acquired within 7 days of witnessed stroke onset, and (5) conservative or intravenous thrombolytic treatment. Among other clinical parameters, patient’s age, sex, ischemic hemisphere side, and severity of neurological deficit (NIHSS) at admission were recorded. Patient datasets were excluded for this secondary study in case of severe imaging or motion artifacts as well as unsuitable post-processing results.

### Imaging

Among others, acute DWI and PWI datasets as well as follow-up FLAIR datasets acquired within 7 days after acute ischemic onset were available and used for this study. MRI with varying sequence parameters was performed with the scanner used for stroke MRI at the admitting hospital of the contributing centers.

DWI acquisition was performed using magnetic field gradient strengths of b = 1000s/mm^2^, averaged for 3–12 directions, and b = 0 s/mm^2^. The in-slice resolution ranged from 0.9×0.9 mm^2^ to 2.0×2.0 mm^2^, while the slice thickness ranged from 6 mm to 7 mm. The in-slice spatial resolution of the PWI datasets also ranged from 0.9×0.9 mm^2^ to 2.0×2.0 mm^2^, while the slice thickness ranged from 6 mm to 6.5 mm. Finally, the in-slice spatial resolution of the follow-up FLAIR datasets ranged from 0.45×0.45 mm^2^ to 1.0×1.0 mm^2^, while the slice thickness ranged from 6 mm to 7 mm.

### Image processing

Basic processing of the DWI, PWI, and FLAIR sequences was performed using the in-house developed software tool AnToNIa [11], briefly summarized in the following. First, apparent diffusion coefficient (ADC) maps were generated based on the DWI sequence and subsequently used for segmentation of the brain tissue and cerebrospinal fluid (CSF). The brain segmentation was separated into the ipsi- and contralateral hemisphere using the hemispheric fissure approximated by a plane in 3D space constructed based on two manually defined lines in distant axial slices. The PWI sequence for each patient was corrected for in-slice motion in a first preprocessing step. A slice-time correction and temporal interpolation to 1 second per frame was applied followed by a conversion of the signal curves to concentration time curves. The arterial input function was automatically extracted for each patient using an atlas-based approach. Therefore, the Montreal Neurological Institute (MNI) brain atlas is registered to the average baseline perfusion image using an affine registration. After this, a segmentation in MNI reference space that contains typical locations of the MCA and ICA including a safety margin, which were determined using a previously generated statistical cerebrovascular atlas [12], is transformed to the average baseline perfusion image. The corresponding concentration time curves within this segmentation are separated into arterial and non-arterial signals using a k-means clustering approach, whereas the concentration time curves from the cluster with the earlier time-to-peak and higher peak are averaged to a final arterial input function using a geometrically correct method. All automatically identified arterial input functions were manually checked for proper quality. The extracted arterial input function for each dataset was then used for calculation of the perfusion parameter maps (CBF: cerebral blood flow, CBV: cerebral blood volume, MTT: mean transit time, and Tmax: time to maximum of the residue function) using a block-circulant deconvolution approach applying a threshold of 0.15 [13]. Two neuroradiologists segmented the infarct lesions in the follow-up FLAIR datasets in consensus.

Afterwards, the perfusion parameter maps (CBF, CBV, MTT, Tmax) and the segmented follow-up lesions were registered to the DWI (B0) image. The contralateral brain segmentation, excluding the CSF segmentation, was then used to calculate average values of the perfusion parameters, which were further used for the normalization of the perfusion parameter maps (subtraction for MTT and Tmax, and ratio for CBF and CBV).

For the CBF and CBV parameters, the magnitude of the measurement denotes an important property of the brain tissue so that the ratio compared to the unaffected hemisphere is a meaningful measurement. For temporal parameters, the magnitude of the measurements can be affected by multiple aspects, including differences regarding the contrast injection protocol and the cardiac output function so that the difference in time between the affected and unaffected hemispheres is most informative. Thus, subtraction was used for the normalization of the temporal MTT and Tmax parameters.

In order to incorporate comparable spatial information within the prediction models, each DWI (B0) image was non-linearly registered to the Montreal Neurological Institute (MNI) brain atlas in the next step using the software package ANTs, whereas the resulting transformation was used to transform all parameter maps (ADC and normalized perfusion parameters) as well as the corresponding CSF and follow-up lesion segmentations into the MNI reference space [14]. A concatenation of the calculated transformations was used for this in all cases to prevent doubling of interpolation errors.

Visual quality checks were performed in each processing step. Patients with relevant artifacts in the image data, low image quality, no contrast agent in PWI sequence, suboptimal registration quality, or no visible follow-up lesions were excluded from further analysis.

### Machine learning models

Three different machine learning models, including logistic regression (LR) [15], random forest (RF) [16], and XGBoost (XGB) [17], were employed in this work to predict the voxel-wise infarct outcome based on the described voxel-wise diffusion and perfusion features as well as spatial information. While LR was introduced quite early in the field of tissue outcome prediction [6], RF and XGB were introduced to the field rather recently [8, 18].

LR models the relation of the expected value of a binary variable *y* depending on several independent variables *x*_1_, *x*_2_, …, *x*_*n*_ through the equation $E(y|x_{1},x_{2},\ldots ,\;x_{n})=\frac{1}{1+e^{{-(\beta }_{0}+\beta_{1}x_{1}+\cdots +\beta_{n}x_{n})}}$. This turns out to represent the probability of a voxel to develop a lesion when *y* encodes the voxel outcome as 1 (lesion) and 0 (non-lesion). LR is known to be comparably fast and its fitted *β*-coefficients allow investigating the impact of changes in the *x* variables on the outcome probability. However, the main limitation of this model is that it is not suitable to model inherently nonlinear relationships.

RF consists of a collection of randomly grown decision trees, which are averaged to a final prediction. As each tree gets trained independently, the training process is easily parallelizable. RF requires defining several hyperparameters, such as the number of trees, their depth, and the amount of training data randomly selected for training of the individual trees. Decreasing the latter often leads to lower correlations between individual trees. Single decision trees are typically considered very sensitive to noise in the data and prone to overfitting. However, the aggregation of several uncorrelated trees is typically considered more robust and generalizable to new data. In contrast to LR, RF is able to capture non-linear relationships and interactions in the training data.

XGB is another tree-based ensemble method, which adds some sophisticated features to the typical gradient boosting machine (GBM) implementation. A GBM ensembles several weak learners, *e*.*g*. decision trees, in an iterative manner. Before each round of boosting, the GBM updates the weights of the training data according to the residuals of the aggregated predictions from previous rounds. While GBM adds one tree at each stage to optimize the overall performance (represented by an objective function), XGB most notably adds a regularization term to the objective function, which penalizes complex trees and leads to less complex weak learners. Therefore, XGB is considered less prone to overfitting. The learning process can be controlled by a rather large set of hyperparameters, which allow to regulate the overall bias-variance trade-off. In recent years, XGB received a lot of attention and was used in many machine learning models winning competitions while outperforming other machine learning techniques on tabular data regarding training time. More details about its implementation and optimizations are described by Chen et al. [17].

### Spatial features

Two different spatial features were investigated in this work with respect to the potential to improve the prediction accuracy. More precisely, all available ADC and normalized perfusion (CBF, CBV, MTT, and Tmax) features were used for model training and testing as well as the MNI reference atlas space coordinates (x, y, z) and voxel-wise lesion probabilities. For including the MNI coordinates efficiently, the x-axis was shifted to the middle of the MNI space so that the hemispheric fissure of the MNI brain atlas is located at x = 0. This axis shift allows to differentiate hemispheres by the sign of the x-coordinate. As only unilateral strokes were included in this study, voxels from the contralateral hemispheres were excluded for the tissue outcome prediction in all cases. As the symmetric MNI brain atlas was used in this work, MNI positions with the same absolute values of x, y, and z were treated as the same position. Therefore, no differentiation between hemispheres was made.

Patient-specific lesion probability maps were generated to prevent a double dipping in the following evaluation procedure. Therefore, the relative frequency of lesions at each voxel position was calculated for each dataset using the other 98 datasets. In doing so, the lesion information from the dataset the lesion probability map was generated for was not included in this calculation. Thus, double dipping is prevented in the cross-validation scheme used for evaluation.

### Machine learning setups

The following four feature combinations were investigated for each machine learning model described above:

1. DWI (ADC) and PWI (CBF, CBV, MTT and Tmax) features
2. DWI and PWI features, and MNI coordinates
3. DWI and PWI features, and voxel-wise lesion probabilities
4. DWI and PWI features, MNI coordinates, and voxel-wise lesion probabilities

As XGB is a high-level machine learning model with several hyperparameters and a relatively low overall training time, a total of seven parameter settings, randomly selected from a predefined equidistant parameter value grid, were evaluated (see appendix). Therefore, 36 different models were trained in total (4 LR-, 4 RF-, and 28 XGB models). Each RF was trained with 100 trees and 60% of random training data per tree. For the training of all machine learning models, the number of non-lesioned voxels used for machine learning model training was restricted to the number of lesioned voxels using random sampling in the ipsilateral hemisphere for each patient, as imbalanced training sets often lead to worse results compared to balanced training sets [19]. All tissue outcome predictions were generated in R (version 3.4.2) [20].

### Evaluation

All lesion outcome prediction models were evaluated using the area under the receiver operating characteristic curve (ROC AUC) [21] and the Dice similarity metric [22] using a ‘leave-one-patient-out’ cross-validation approach. In doing so, the tissue outcome for each patient was predicted and evaluated without incorporating any data from the regarding patient within the model training process, which also includes the lesion probability map. All machine learning models described above generate likelihood maps for tissue infarction. The ROC AUC reflects the suitability of the likelihood score to discriminate between the two outcome classes independently of a specific threshold. It can also be interpreted as the probability that a randomly chosen infarct voxel receives a higher risk than a randomly chosen non-infarct voxel. Hence, a ROC AUC of 1.0 would describe a perfect model, while a ROC AUC of 0.5 would indicate that the model is not advantageous compared to random guessing. In contrast to this, the likelihood maps have to be binarized for the calculation of the Dice similarity metric. However, the likelihood calculation is not comparable between models so that a single threshold (*e*.*g*. 50%) might not lead to optimal results across all models. Thus, the probability threshold needs to be optimized for every model, setup, and cross-validation run using the training data. Therefore, all lesion prediction maps were first generated using the cross-validation scheme described above. Afterwards, the optimal threshold leading to the overall best average Dice score for the actual lesion prediction was determined also using cross-validation principles. More precisely, the patient-individual optimal threshold for lesion probability binarization was determined by identifying the threshold that maximizes the Dice coefficient using all lesion predictions and corresponding true follow-up lesions from all other training datasets. Then, the optimal threshold was applied for binarizing the tissue outcome prediction on the test patient and its corresponding Dice coefficient was calculated. After calculating the ROC AUC and Dice coefficient for each patient within the cross-validation, the averages and standard deviations of these metrics were calculated for each model setup.

## Results

### Patient characteristics

Table 1 shows the characteristics of the 99 patients included in this study. Overall, the patients from the different centers were comparable in terms of age, the median NIHSS, gender distribution, and affected hemisphere. Contrary to this, the treatment distribution was different among the centers and the patients from two centers showed considerably smaller follow-up lesion volumes but also contained the smallest patient samples among the contributing centers.

**Table 1:** Characteristics of the 99 included patients included in this study.

Origin	N	Left/right	IV-tPA/no tPA	Follow-up lesion volume (in ml)	Male/female	Age	NIHSS
Center 1	12	6/6	12/0	16.49 (±30.67)	7/5	65.2 (±7.9)	9.08 (±5.3)
Center 2	2	0/2	2/0	7.17 (±1.02)	1/1	74.5 (±5)	8.5 (±5)
Center 3	28	15/13	14/14	36.99 (±87.25)	14/14	68.7 (±12.1)	12.6 (±6.1)
Center 4	19	11/8	16/3	27.98 (±49.23)	10/9	70 (±12.5)	9.12 (±4.1)
Center 5	38	24/14	19/19	31.62 (±93.48)	23/15	64 (±14.1)	11.2 (±6.6)
All	99	56/43	63/36	28.62 (±79.26)	55/44	66.8 (±12.6)	10.9 (±6)

### Lesion prediction results

Table 2 shows the quantitative results of the lesion prediction models using the ROC AUC and Dice metrics. Overall, two XGB models, which included spatial information, performed best regarding the average ROC AUC and Dice values, achieving a ROC AUC of 0.89±0.09 (setting 3 using the MNI coordinates) and a Dice coefficient of 0.395±0.229 (setting 7 using the lesion probabilities). The latter setting also achieved the highest average of the ROC AUC and Dice coefficient. Both models revealed highly significant improvements (p < 0.01) compared to any other model without spatial information—regarding both metrics and effect sizes of 0.064 (ROC AUC) and 0.048 (Dice) compared to the best models without spatial information.

**Table 2:** Average ROC AUC and Dice results from the leave-one-patient-out cross-validations for each model.

Model	Features	Setting	mean ROC AUC	mean Dice	training time (s)
LR	ADC + PWI		0.813±0.107**	0.317±0.220**	41
LR	ADC + PWI + MNI		0.827±0.100**	0.292±0.229**	48
LR	ADC + PWI + LP		0.874±0.108**	0.319±0.238**	45
LR	ADC + PWI + MNI + LP		0.877±0.099**	0.322±0.232**	44
RF	ADC + PWI		0.826±0.104**	0.341±0.218**	614
RF	ADC + PWI + MNI		0.891±0.092	0.383±0.226**	628
RF	ADC + PWI + LP		0.883±0.104**	0.371±0.227**	622
RF	ADC + PWI + MNI + LP		0.889±0.092	0.368±0.228**	758
XGB	ADC + PWI	7	0.830±0.105**	0.346±0.220**	77
XGB	ADC + PWI + MNI	3	**0.893±0.085**	0.387±0.213	179
XGB	ADC + PWI + LP	7	0.888±0.101	**0.395±0.229**	95
XGB	ADC + PWI + MNI + LP	6	0.887±0.098*	0.386±0.224	157

Best results according to each metric are highlighted in bold text. Significant differences to this best-performing method computed with a one-sided paired student’s t-test are marked with a star (*) for a confidence interval of 95% (p < 0.05) and two stars (**) for a confidence interval of 99% (p < 0.01). Nominal p-values are reported without correction for multiplicity, similarly as in [23]. For the XGB models, the best setting for each feature combination in terms of average of ROC AUC and Dice metric was used. The full table including all XGB results can be found in the online supplement. Training time refers to the time required to train one machine learning model on the whole dataset. ADC = apparent diffusion coefficient, PWI = perfusion-weighted MRI parameters, MNI = MNI coordinates, LP = lesion probability.

**Fig 1:**
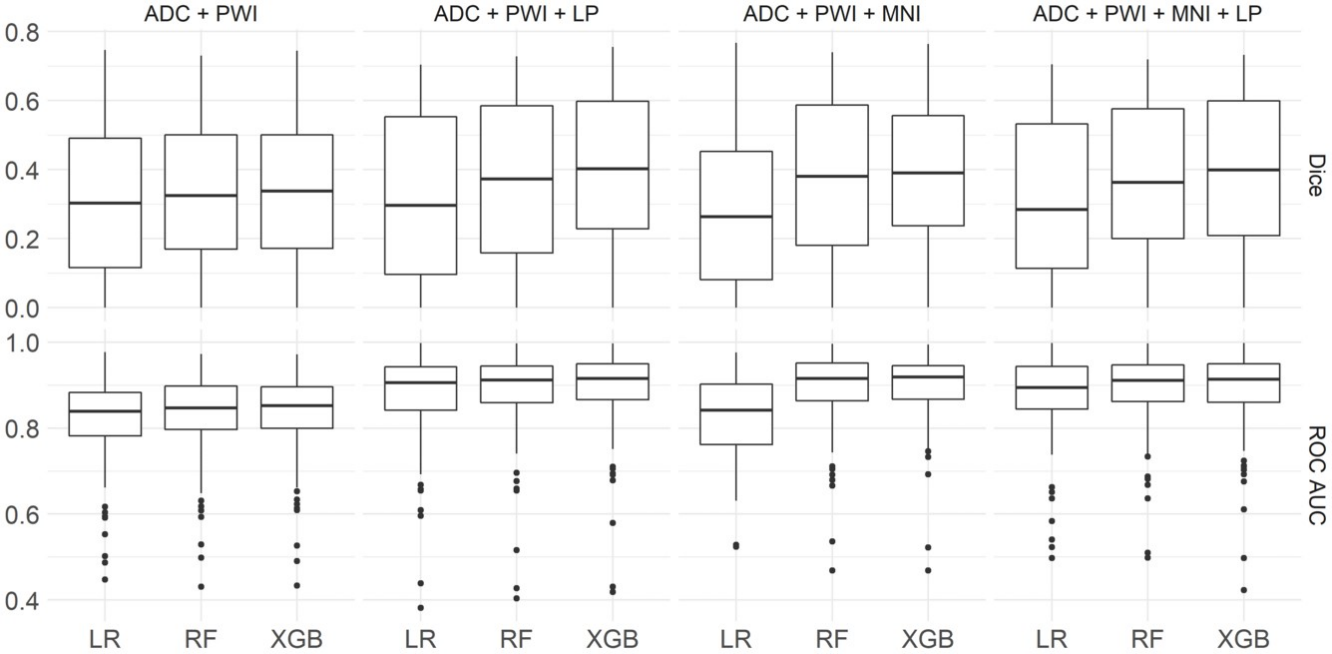
Model performances. Boxplots representing ROC AUC and Dice coefficients for each feature combination for all LR, RF, and the best XGB models.

**Fig 2:**
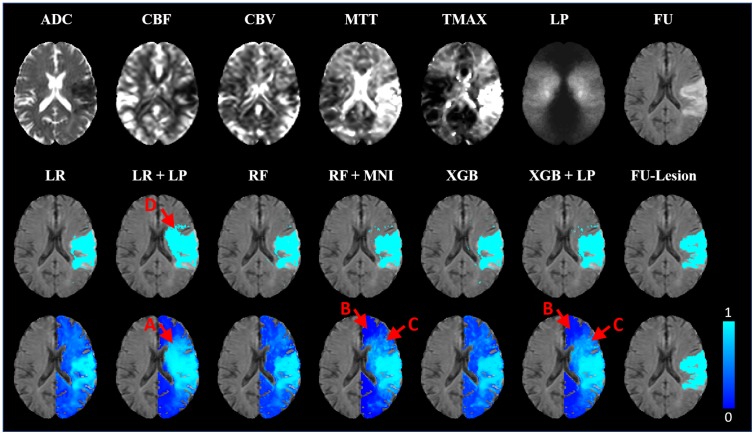
Prediction example for a selected patient. Imaging parameters, lesion probability map (LP), and true follow-up FLAIR datasets (top row), and predictions represented by binary infarct prediction (middle row) and lesions likelihood maps (bottom row) for models without and best models with spatial information, as well as the corresponding final follow-up lesion for a selected patient. For this patient, all binarized prediction maps, except those generated using LR including the lesion probability (A), correspond well with the final infarct outcome. The prediction maps mostly differ in the spatial distribution of voxels with lower (B) to medium (C) infarct risk. While these seem more randomly distributed for models containing only ADC and PWI parameters, the low risk areas (B) are concentrated in areas of low lesion probabilities and medium risk areas (C) are concentrated in areas of high lesion probabilities for models containing spatial information. However, this leads to smoother lesion risk prediction maps and the infarcted areas remain clearly distinguishable in case of RF and XGB. In case of LR, a strong overestimation of the infarct occurs in the binarized map (D).

The XGB models with the highest average ROC AUC and Dice coefficients per feature combination (see Table 2) were investigated for further analysis. In general, these XGB and all RF models performed significantly better (p < 0.001) regarding both metrics compared to the LR model using the respective feature setup. Significant benefits of XGB over RF (p < 0.01) for both metrics were only obtained for the feature combination ADC, PWI, and lesion probability. For the feature combinations ADC and PWI (ROC AUC) as well as ADC, PWI, MNI, and lesion probability (Dice), significant improvements were only found for one metric. Furthermore, model specific comparisons between feature settings with and without spatial features revealed significant performance improvements when incorporating spatial information for all XGB (p < 0.0001) and RF (p < 0.01) models (Fig 1). For LR, spatial features improved the performance in all cases except when MNI coordinates were used exclusively, which even had a negative effect on the Dice coefficient. Performance improvements in ROC AUC were significant (p < 0.00001) for both settings containing the lesion probability. An illustration of the final infarct prediction for models without and best models with spatial information for a selected patient can be found in Fig 2.

### Contribution of features for final infarct prediction—Gain

One of the properties of XGB, which is very convenient, is that it is possible to determine the importance of features rather easily. Therefore, the relative contribution of different features to the best XGB models was determined based on the Gain per feature, which corresponds to the average training loss reduction gained when using a feature for splitting during the training process. The Gain has the advantageous property that its sum over all features is equal to one.

**Fig 3:**
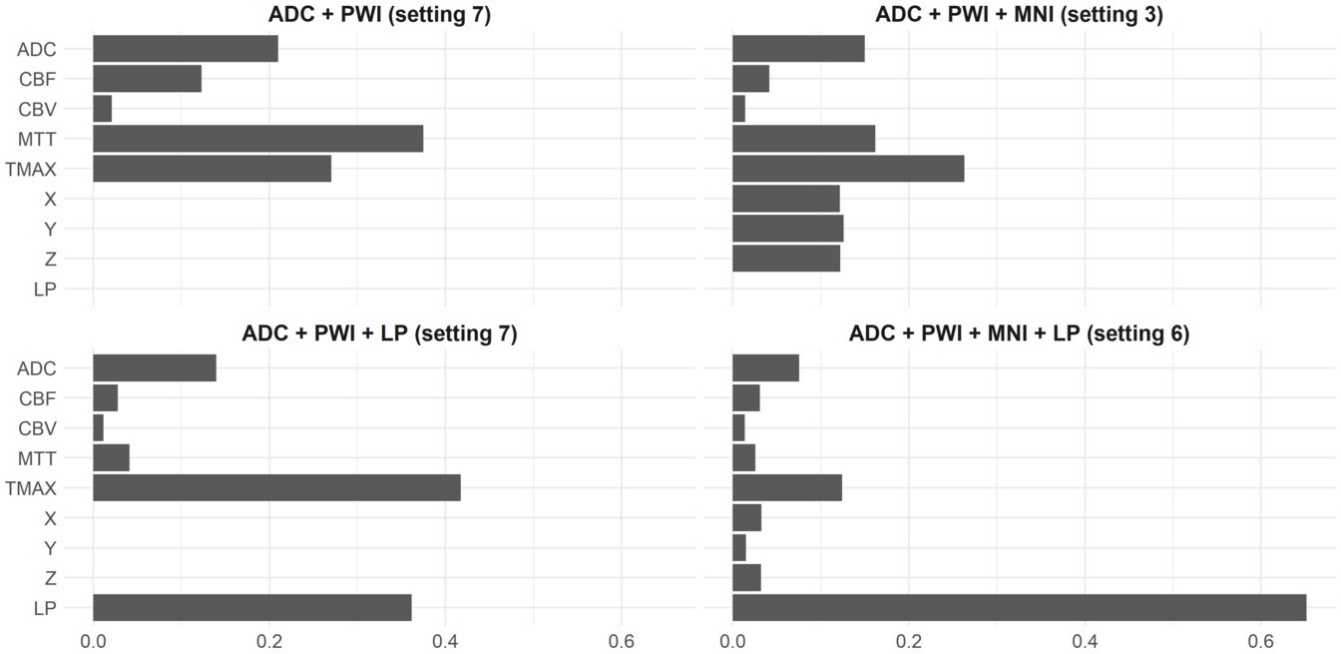
Gain per feature. Gain per feature (in %) of the best XGB model settings for each feature combination analyzed.

As shown in Fig 3, the ADC feature contributes almost a constant average of 14.4% (±5.5%) across all input feature setups. The Tmax and MTT perfusion parameters were found to result in the highest Gains regarding the perfusion parameters. Tmax outperformed MTT in the three settings that included spatial features while MTT had a considerably smaller Gain value in the two models that included the lesion probability feature. CBF and CBV were found to have a rather low impact that decreased even further with the inclusion of spatial features. Generally, the specific contribution of input features was found to be highly dependent on the number of features used for the outcome prediction. However, Tmax and MTT vary especially strong with standard deviations of 12% (Tmax) and 16.1% (MTT).

For the feature combination DWI, PWI, and MNI coordinates, the x, y, and z coordinates contribute a nearly equal amount of 12% Gain each, which sums up to over a third of the overall Gain (37%). Similarly, in the absence of MNI coordinates, the lesion probability map contributes 36.2%. Finally, when adding the MNI coordinates and the lesion probability features together, the lesion probability contributes significantly more than any other feature (65.2%) to the overall Gain. In contrast, the x, y, z MNI coordinates lose a lot of their impact and contribute only a total of 8% (x = 3.3%, y = 3.2%, and z = 1.5%).

### Contribution of features for final infarct prediction—Shapley values

**Fig 4:**
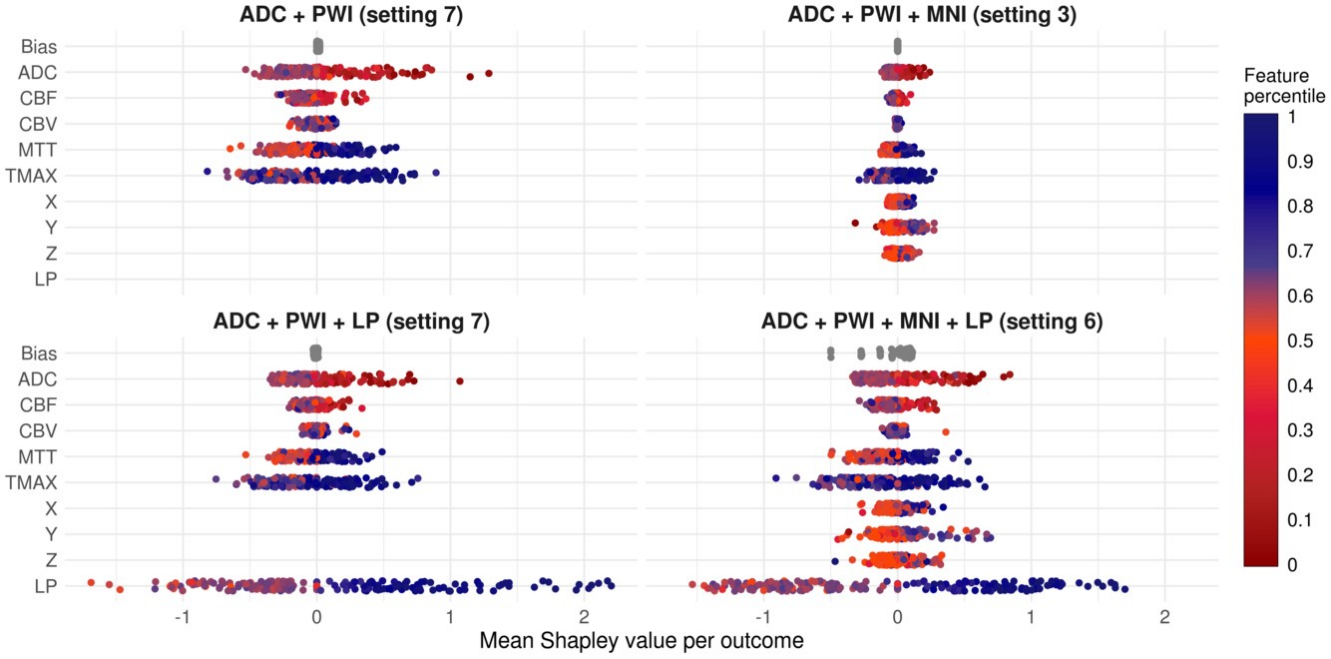
Mean Shapley values and corresponding mean feature percentiles for both voxel outcomes. Mean Shapley values (x-axis) and mean feature values in terms of percentiles (color encoding) for the best XGB models (per feature combination). The means were calculated for both outcomes (lesion yes/no). As the bias is a constant term per model, it only varies between the cross-validation folds and is the same for lesion and non-lesion voxels in each cross-validation run. The color encoding of the dots represents the means of the corresponding feature values (in lesion or non-lesion voxels) in terms of their percentile in the dataset, *e*.*g*. the blue dots in the positive ranges of the Shapley values for the lesion probability (LP) indicate that corresponding LP feature values were relatively high on average in terms of LP percentiles in the underlying datasets. (Positive Shapley values indicate a positive correlation between the respective feature and the infarct risk prediction. However, the impact on the final prediction is not linear, as XGB calculates Shapley values on the logit scale).

Shapley values were introduced in 1953 by Lloyd Stowell Shapley in the field of cooperative game theory. They provide a concept to divide a reward among a coalition of cooperative players. Each player receives an amount of the reward according to his contribution to the surplus generated by the coalition [24]. The concept has been recently transferred to machine learning by Lundberg and Su-In Lee [25]. Here, the role of the reward (including the surplus of the coalition) corresponds to a prediction, players correspond to features, and a player’s contribution corresponds to the value of a feature. Therefore, Shapley values provide a concept to decompose the output of a machine learning model into contributions from its single features. They can be interpreted in similar manner as a feature coefficient by its feature value in the context of a linear model. The Gain was employed to measure the contribution of each feature during training. To determine the impact of the features and their values on new predictions, Shapley values were calculated for each prediction of the four best XGB models considering each feature combination (see Fig 4).

**Fig 5:**
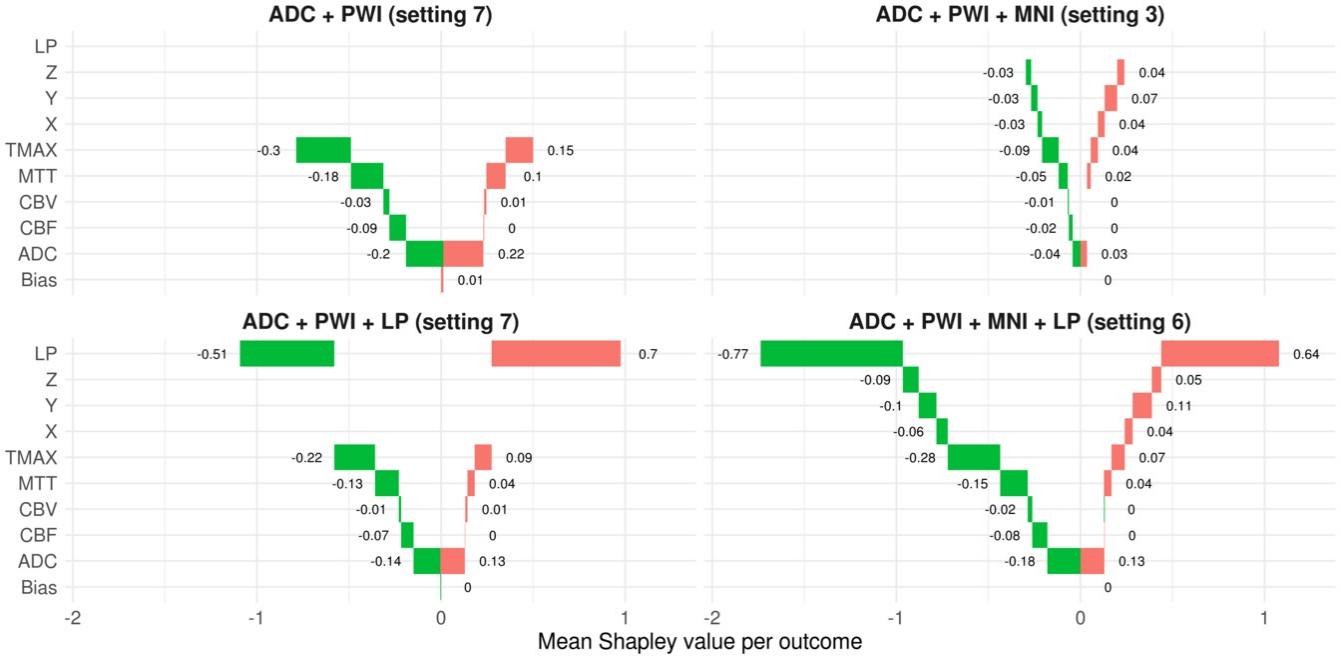
Waterfall breakdown of mean Shapley values per outcome and model. Green bars represent average Shapley values of predictions for voxels with non-lesion outcome. Thus, the sum over all green bars corresponds to the average out of sample prediction score (on the logit scale) for non-lesion voxels. Likewise, the red bars correspond to average Shapley values of predictions for voxels with a lesion outcome.

Shapley values possess some desirable properties and are often considered as state of the art regarding recent debates about interpreting black box machine learning models. One of these properties is that the sum of the Shapley values of all features for one voxel (including the bias) corresponds to the model’s infarct risk prediction at this voxel (on the logit scale). This allows plotting the average feature contribution to the predictions of lesion and non-lesion outcome voxels for each feature, see Fig 5.

**Fig 6:**
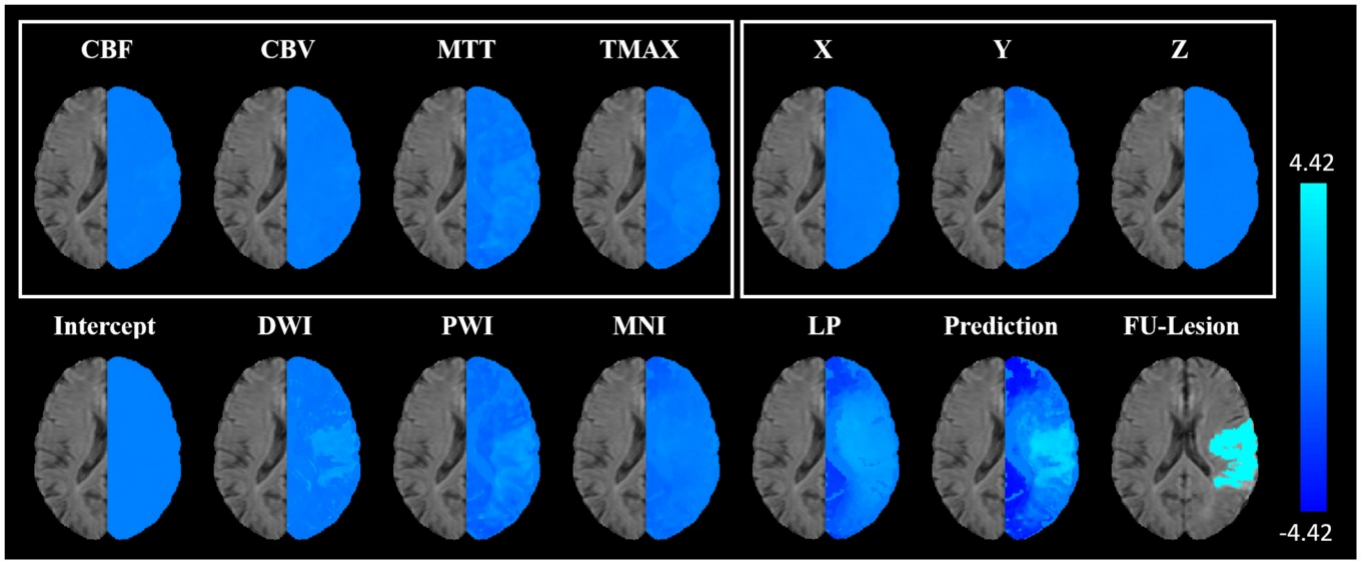
Shapley value decomposition. Shapley value decomposition of the predictions from the XGB model incorporating DWI, PWI, MNI, and LP (setting 6). The sum of the intercept and the Shapley values for DWI, PWI, MNI, and LP correspond to the prediction of the follow-up lesion on the logit scale (bottom row). The Shapley values for PWI and MNI are themselves sums of the Shapley values of CBF, CBV, MTT, Tmax (PWI) and X, Y, Z (MNI; top row). Further addition of the MNI and LP Shapley value maps would lead to a high-level spatial Shapley value map incorporating all spatial features from the model. Similar Shapley value maps for the other three best XGB models per setting can be found in the online supplement.

As Shapley values for several features can simply be added, the individual predictions can be decomposed into contributions of each feature (see Fig 6).

### Contribution of features for final infarct prediction—Permutations

**Fig 7:**
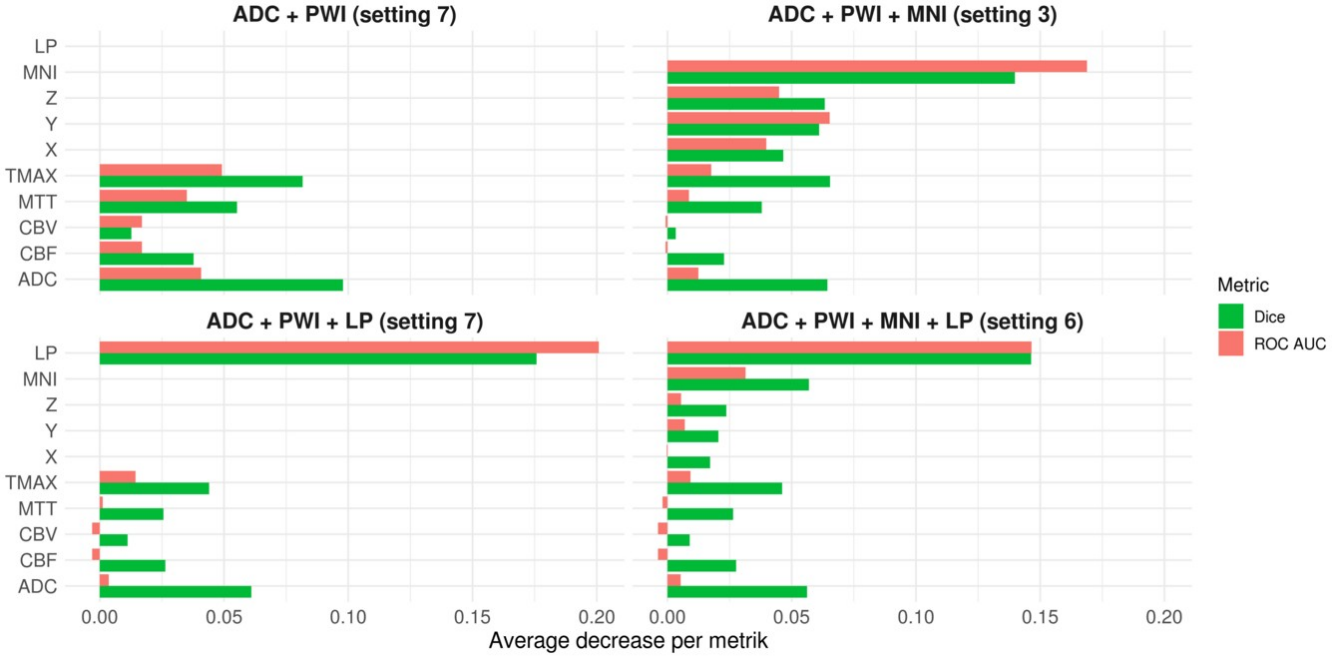
Permutation importance per feature. Average absolute decrease in Dice and ROC AUC metrics for independent permutations of each feature. For the calculation of the Dice coefficients, the same approach for determining the optimal threshold for binarization of the prediction maps as described in the methods section was employed. As simple permutation of only one of the x, y, or z coordinates may lead to individual coordinates that have not been seen by the model during training, additionally x, y, and z coordinates were supplied in combination that resulted from permutations of MNI coordinates as single coordinate points.

In the previous two subsections, the global feature contributions during the training of the best XGB models were investigated in terms of Gain and Shapley values to determine feature contributions for each prediction individually. To determine the contribution of each feature in terms of Dice and ROC AUC during the cross validation, random permutations were applied to each of the model’s features on the out of fold data—one feature at a time. Thus, the regarding feature became uninformative for prediction and the change in the average Dice coefficient and ROC AUC compared to a model with fully informative input can be associated with the loss in information carried by the original feature (see Fig 7. The basic idea to measure permutation importance for features goes back to Breiman [16].)

### Contribution of features for final infarct prediction—Summary

To compare the contribution of the features to the final infarct predictions across models and measures (Gain, Shapley values, and decrease in ROC AUCs and Dice coefficients after feature permutation)–with respect to the four best XGB models—the following procedure was applied. First, the contribution of the MNI coordinates (for Gain and Shapley values) was aggregated by adding them up to a combined MNI feature. In case of Gain, this is considering that the information provided by the coordinates is literally orthogonal and also supplementary. Therefore, the paths of the trees in the XGB models arguably contain splits among several MNI dimensions. For Shapley values addition occurs just naturally as showcased above. In case of the permutation measures, the aggregation was not needed as the contribution of the MNI coordinates was explicitly evaluated via joint permutation of the coordinates. Afterwards, four rankings—one per metric—were obtained for each model. In case of Shapley values, features were ordered by the average difference between Shapley values in lesion and non-lesion voxels (see Fig 5). Finally, the rankings were normalized according to the number of features in each model and evenly scaled between 0 (lowest rank) and 1 (highest rank).

In summary, the spatial lesion probability (LP) feature achieved the highest ranking-score (0.975) and was the top feature among both models that included it, only exceeded in terms of average Gain by Tmax for the DWI-PWI-LP model. The MNI coordinates (0.896), contributed most to the DWI-PWI-MNI model and third most to the DWI-PWI-MNI-LP model where it placed behind LP and Tmax—regarding the average Gain. Tmax had the third highest average ranking score (0.785) and achieved the highest rank among the PWI features. These were mostly ranked in the order Tmax—MTT (0.442)—CBF (0.215)–CBV (0), except for the Gain-rankings of the DWI-PWI model, where Tmax ranked behind MTT and the DWI-PWI-MNI-LP model, where CBF was ranked ahead of MTT. The fourth highest average rank was achieved by ADC (0.623). It was always ranked above CBF and CBV. Permutations of ADC resulted in the second highest decrease in the Dice coefficient ranked after the spatial features and, in case of the DWI-PWI-MNI model also after Tmax. Furthermore, it contributed third most in terms of ROC AUC permutation importance and Shapley values, following the spatial features and Tmax. The only feature besides the spatial ones and Tmax which ranked higher than ADC, was MTT in the Gain-rankings of the DWI-PWI and DWI-PWI-MNI models.

## Discussion

### Tissue outcome prediction

The main finding of this work is that advanced non-linear machine learning models including spatial information as features perform superior predicting tissue outcome in acute ischemic stroke patients compared to the corresponding models that do not include spatial information. Only in case of the linear model, the exclusive inclusion of MNI coordinates without the spatial lesion probability information led to a decrease of the average Dice coefficient. Furthermore, the tree-based XGB and RF models achieved competitive results regarding the ROC AUC and Dice metrics, clearly outperforming the linear model.

### Spatial features in linear models

Considering the inhomogeneity and complexity of the brain, it does not seem reasonable to assume a simple linear relation between the different aspects of brain physiology and the spatial infarct risk. Thus, locally varying infarct risk can only be incorporated in a linear model by the lesion probability mask but not by Cartesian coordinates within the brain. Linear models have their strengths in modelling datasets consisting of (possibly sparse) categorical and continuous features, which are monotonous (at least under transformations). Thus, it is expected that raw MNI coordinates, which describe a linear direction within the brain, have no practical use for the outcome prediction and even lead to decreased accuracy with respect to the Dice score if used without the lesion probability information. However, as there seems to be some pattern in the overall dataset regarding the spatial occurrence of infarcts as can be seen in the lesion probability map (Fig 2), a small ROC AUC increase of 1.3% was observed. This rise in the ROC AUC compared to the simultaneous decrease of 2.5% in the Dice coefficient results from a better detection of correctly classified non-lesion voxels (true negatives), as these represent most of the brain and are not taken into account for the Dice coefficient calculation.

In contrast to MNI coordinates, the sole incorporation of the lesion probability led to a significant increase of the ROC AUC (6.09%) and a nearly equal Dice coefficient. Since a continuous, monotonous relation between positional infarct probability in the training data and the test data is to be expected, the lesion probability is much easier to incorporate for the linear model compared to the MNI coordinates. Consistently, the incorporation of the MNI coordinates in addition to the lesion probability did not lead to any further significant improvement of the evaluation metrics. However, apart from quantitative improvements in the evaluation metrics, visual checks revealed that high infarct risk probabilities in individual patients often correspond to areas that also show high lesion probabilities in the population-based lesion probability map independent of the other imaging features. This unwanted correspondence led to considerable over- and under-estimations of the predicted lesion compared to other models used in this study (Fig 2). As there is no evidence for direct causation between population-based lesion probabilities and the infarct outcome in the individual patient, the linear model clearly lacks the interaction of the lesion probability feature and the imaging parameters, which are known for their causal relation to the infarct outcome. Therefore, this model is likely to be not generalizable to cases with differing spatial distributions of the infarct areas.

### Spatial features in tree models

In contrast to the linear model, the results suggest that the XGB and random forest models are capable of handling MNI coordinates directly in a meaningful manner. These models consist of iteratively (XGB) or parallel (random forest) trained tree models that split features several times. Therefore, the ensemble trees are very robust handling categorical features with fixed level-ranges and non-monotonous, continuous variables, which might contain several local maxima. Applying such trees to a feature set containing the MNI coordinates possibly leads to several splits along the x, y, and z direction in MNI space. Each of these split points is basically a two-dimensional hyperplane in the MNI space. Combining all these hyperplanes will fragment the MNI space into multiple small areas. As the individual trees map different risk probabilities to each side of the spatial splits, each parcel provides an accumulated different ‘basic’ infarct risk. Therefore, the combination of these parcels provides a rough infarct probability map with spatial perfusion thresholds, learned from the data. The granularity of these parcels is most likely lower compared to a voxel-wise lesion probability map. However, in principle, this is only restricted by the variety of the infarct prevalence along the spatial dimensions in the training data and the hyperparameter setting selected for the training of the regarding tree ensemble. The similar results for the XGB and RF settings, containing the MNI coordinates or the lesion probability information, suggest that both spatial features capture mostly similar spatially dependent information. In contrast to the logistic regression, these models also did not reveal any considerable issues regarding over- or underestimation of the lesion size in areas of high or low lesion probabilities. This might be the case because both tree ensembles are able to automatically identify interactions between the spatial and imaging features in the training data. Therefore, a high or low lesion probability might not substantially affect the prediction in individual patients as the regarding MNI coordinates or lesion probability dependent splits are just parts of a path in a tree, which typically contains further splits of the ADC and perfusion parameters.

### Contribution of features for final infarct prediction in XGB models

As consistently indicated by the rankings regarding the average Gain per feature, the differences in the average Shapley values for lesion and non-lesion values, and the permutation importance measured by the decrease in ROC AUCs and Dice coefficients for random permutations of one feature at a time during the cross-validation, the spatial features almost always exhibited the highest contribution. While the magnitude of lesion probability contribution was comparatively high and always ranked higher than the contribution of the (aggregated) MNI coordinates, the information encoded in these two spatial features seems to be comparable. This is supported by the comparable cross-validation results regarding ROC AUC and Dice coefficient, the theoretical aspects mentioned in the last paragraph as well as the visual inspections of the Shapley value maps for MNI and the lesion probability in the DWI-PWI-MNI and the DWI-PWI-LP models (see S1 Fig). However, as the distributions of both Shapley value maps seem to be highly correlated with the lesion probability map (see Fig 2 for visual inspection) and considering the high contribution of the spatial features, generalization of the spatial models to new datasets should be conducted with care. The high magnitudes of the lesion probability and the MNI coordinate contributions might raise the concern that interactions between spatial and PWI/DWI features possibly learned by the XGB models might not be generalizable if the trained model is applied to cases with strokes in different areas such as the brain stem or posterior flow territory. Thus, it is likely that these models would benefit from the integration of datasets with infarcts in other brain regions and possibly differing occlusion locations.

Apart from the spatial features, the most informative features across all models were Tmax followed by ADC and MTT. While MTT was especially contributing to the high average Gain in the model training phase, permuting ADC revealed comparatively high decreases in the Dice coefficient. Many previous studies have used ADC to determine the infarct core while Tmax and MTT are mostly used to define the penumbra of an acute ischemic stroke. Thus, it is not surprising that these features have a high informative value for tissue outcome prediction. In contrast to these findings, the CBF and CBV features were almost always the least contributing features, which could be explained by the high variation of CBF and CBV in brain tissue (*e*.*g*. white and gray matter difference).

### State of the art—ISLES challenges

To address the challenge of comparability between methods, the Ischemic Stroke Lesion Segmentation challenge (ISLES) was started in 2015 [9]. While the initial challenge was focused on ischemic stroke lesion segmentation, the 2016 and 2017 challenges aimed at lesion outcome prediction after ischemic stroke. During the challenges in 2016 and 2017, 24 teams employed different strategies and methods competing to predict the 90-day follow-up tissue outcomes for 19 (2016) and 32 (2017) patients, respectively. For all competitors, n = 35 (2016) and n = 43 (2017) patient datasets were available to train their algorithms. These datasets included pre-generated ADC, CBF, CBV, MTT, and Tmax maps. Intentionally, only minimal pre-processing steps were uniformly applied to the data. In 2016, mostly predictions based on classical machine learning methods were submitted, while the participants made exclusive usage of deep learning models in 2017, which mostly outperformed previous methods. To the best of our knowledge, only Robben et al. [26] and McKinley et al. [27] incorporated similar spatial features in the form of atlas coordinates, as used in this work. None of the submissions employed extreme gradient boosting. Monitored performance metrics included the precision, sensitivity, Hausdorff distance, average symmetric surface distance, and the Dice coefficient. The overall winning method in 2017 achieved a Dice coefficient of 0.31±0.23, while the best method regarding the Dice metric only achieved a Dice coefficient of 0.32±0.23. The best performing approach (XGB in combination with lesion probability) presented in this work achieved a considerably better average Dice coefficient 0.395±0.229. However, the datasets used for training and evaluation are different so that the results are not directly comparable as, for example, smaller lesions are more challenging to predict and lead to smaller Dice coefficients so that no direct comparison is possible. In order to establish comparability and reproducibility in this research area, all datasets from these challenges remain available online. However, in this research project, no datasets from the ISLES challenges were used because the raw datasets PWI and DWI were not made available and the calculated ADC and perfusion maps were likely processed differently than the datasets used in this work, potentially introducing a systematic bias. For this reason, the choice was made to use a much bigger database to allow improved training of the machine learning models.

Although remarkable progress regarding the quality of lesion outcome prediction and sophistication of employed methods has been achieved in recent years, a general breakthrough has not been reached so far. Even with the increasing amount of available training data, improvements in the prediction results seem to be limited by the complex nature of the problem.

A major conclusion of the ISLES challenge results was that current or new methods should incorporate clinical information as well as *a priori* physiological information on stroke infarction, while keeping the transparency and interpretability of employed methods in mind. The machine learning models described in this work incorporate spatial information in the form of probability maps and MNI coordinates into existing and recently developed multi-parametric machine learning methods, which allow investigating the importance of the various features, thus, addressing these considerations.

### State of the art—Other convolutional networks

Recently, Nielsen et al. proposed a deep convolutional neural network to include spatial information for voxel-wise stroke prediction of 30 days follow-up tissue outcome [7]. A total of nine biomarker maps were used in different convolutional neural network architectures. Using 158 datasets for training and 29 patients for validation, an average ROC AUC of 0.88±0.12 was reported for the scenario, which is in the range of the highest ROC AUC of 0.89±0.09 achieved by the best XGB model in this study (setting 7 incorporating ADC and PWI perfusion features as well as MNI coordinates). In contrast to the smooth prediction images of their method, the prediction results of the models achieved in this work appear more scattered, which might be a result of noise and other imaging artifacts in the data that have a reduced effect if multiple voxels within a neighborhood are analyzed as in convolutional neural networks. However, scattered noise artefacts in the final prediction map can also be removed easily using simple neighborhood analysis methods. In contrast to the reported five days of training for the deep convolutional neural network, the XGB method achieving comparable ROC AUC results took only 95 seconds to train on a conventional computer, which highlights the potential of this machine learning method to experiment with other setups more quickly but also suggest that tree ensemble methods such as random forests or XGB should be also used as a comparison method instead of the simple linear model when evaluating new stroke prediction methods employing convolutional neural networks.

### State of the art—XGB

Despite the highly competitive performance of the XGB algorithm published in 2014, its application in acute stroke tissue outcome prediction is still rare. Only recently, Livne et al. presented a stroke prediction method with a reported ROC AUC of 0.88 achieved using a leave-one-out cross-validation based on 195 patients, from two studies [8]. For modelling, 12 biomarkers were used including normalized T2-FLAIR, DWI, and 10 PWI features. However, no spatial information was used. Hyperparameters were tuned within each training process and the data was sampled to balance the class differences of lesions and non-lesion voxels. The follow-up times were not reported. Regarding the differences to this study, it is speculative to explain the comparably high ROC AUC of 0.88, which was achieved without incorporation of spatial features. One reason might be the evaluation strategy in our work, which did not take into account voxels in the contralateral hemisphere, possibly leading to lower true negative rates and a decrease of the ROC AUC. Unfortunately, no Dice coefficients were reported by Livne et al., which would have shed light on this aspect. However, it seems beneficial to combine the imaging features used in the study by Livne et al. with the spatial features suggested in this work.

### Reproducibility, general applicability, limitations, and outlook

It should be highlighted that the performance of any machine learning model depends on the data used for training and testing. Imaging data usually does not only differ due to the acquisition process, but also due to patient-individual characteristics and stage of the stroke. Within this context, it also needs to be mentioned that the time of follow-up imaging was ranging between 1 and 7 days, which can also influence the prediction accuracy as the lesion change in appearance during this time, for example, due to changes in water accumulation within the lesion. Furthermore, varying steps in the preprocessing pipeline, differing software, and observer differences regarding the segmentation of the final lesion make it hard to compare results between different experimental setups. As the data used in this work was collected in different hospitals with the same study protocol and our preprocessing pipeline contains only standardized analyses and image sequences, the results described in this work should be reproducible using comparable datasets. It should be mentioned that the proposed lesion outcome prediction models were only applied to datasets of patients with first-ever unilateral strokes. Thus, it remains to be evaluated how these models would perform in patients with secondary or bilateral strokes.

However, for machine learning models that are not as robust as tree-based models regarding the properties of raw MNI coordinates, incorporating only lesion probabilities seems suggested, especially since the performance was rather similar. Furthermore, it needs to be highlighted that this is not the first method to employ the MNI coordinates in this domain. Robben et al. [26] and McKinley et al. [27] used these features among other engineered spatial features within the ISLES 2015 and 2016 challenges (http://www.isles-challenge.org/). Both used these features within a modified approach based on (extreme) random forests for ischemic stroke lesion segmentation. However, the contribution of the MNI coordinates within these approaches was not discussed.

It needs to be pointed out that the spatial features used in this work do not account directly for the correlation that can be expected between neighboring voxel and each voxel is still treated independent of the neighborhood. This problem can be partly solved by postprocessing methods, *e*.*g*. applying connected component analyses or Markov random fields [28]. However, further research is needed to directly model spatial correlation in the prediction model. Within this context, it might also be beneficial to include lesion probability maps within a multi-modal deep convolutional neural network approach for tissue outcome prediction. Finally, it needs to be pointed out that the cross-validation used in this work has some disadvantages compared to using a completely independent test set. Although the multi-center sample available for this study is comparably large, it is likely not large enough for training of the machine learning model if the data would have been split into independent training, validation, and test sets. Thus, the generalizability and reproducibility of the proposed machine learning models incorporating spatial features should be investigated in more detail using an independent test set in future.

In conclusion, the incorporation of spatial information can lead to significant performance improvement for tissue outcome prediction of patients suffering from acute stroke.

## Supporting information

S1 Table Different parameter settings for the XGB models. Apart from these differences, each model was run with the following similar properties: objective = binary: logistic; booster = gbtree. An up-to-date description of the exact role of each parameter and its possible values is provided in https://xgboost.readthedocs.io/en/latest/parameter.html.
(DOCX)

S2 Table Average ROC AUC and Dice results from the leave-one-patient-out cross-validations for each model. Best results according to each metric are marked in bold text. Significant differences to this best-performing method computed with a one-sided paired student’s t-test are marked with a star (*) for a confidence interval of 95% (p < 0.05) and two stars (**) for a confidence interval of 99% (p < 0.01). Nominal p-values are reported without correction for multiplicity, similarly as in [23]. ADC = apparent diffusion coefficient, PWI = perfusion-weighted MRI parameters, MNI = MNI coordinates, LP = lesion probability.
(DOCX)

S1 Fig Shapley value decomposition maps. Shapley value decomposition maps of the predictions from the best XGB models incorporating DWI and PWI (setting 7; rows 1–2), DWI, PWI, and MNI coordinates (setting 3; rows 3–4), DWI, PWI and LP (setting 7; rows 5–6).
(DOCX)
