# Correction: Improved multi-parametric prediction of tissue outcome in acute ischemic stroke patients using spatial features

**DOI:** 10.1371/journal.pone.0230653

**Published:** 2020-03-12

**Authors:** Malte Grosser, Susanne Gellißen, Patrick Borchert, Jan Sedlacik, Jawed Nawabi, Jens Fiehler, Nils Daniel Forkert

The image for S1 Fig, “Shapley value decomposition maps,” does not appear. Please view the complete S1 Fig here.

## Supporting information

S1 Fig Shapley value decomposition maps. Shapley value decomposition maps of the predictions from the best XGB models incorporating DWI and PWI (setting 7; rows 1–2), DWI, PWI, and MNI coordinates (setting 3; rows 3–4), DWI, PWI and LP (setting 7; rows 5–6).
(TIF)
