# Supplementary material for: Improved multi-parametric prediction of tissue outcome in acute ischemic stroke patients using spatial features
Source: PLoS One. 2020 Jan 24;15(1):e0228113. doi: 10.1371/journal.pone.0228113 (PMC6980585; doi:10.1371/journal.pone.0228113)
Supplement: S1 Table — Apart from these differences, each model was run with the following similar properties: objective = binary: logistic; booster = gbtree. An up-to-date description of the exact role of each parameter and its possible values is provided in https://xgboost.readthedocs.io/en/latest/parameter.html. (DOCX) [file pone.0228113.s001.docx]

**S1 Table.** **Different parameter settings for the XGB models.**

| Setting | eta | min_child_weight | subsample | colsample_bytree | max_depth | gamma | base_score |
| --- | --- | --- | --- | --- | --- | --- | --- |
| 1 | 0.05 | 4 | 0.3 | 0.05 | 5 | 0.90 | 0.25 |
| 2 | 0.75 | 2 | 0.7 | 0.45 | 11 | 0.34 | 0.75 |
| 3 | 0.05 | 2 | 0.1 | 0.65 | 14 | 0.18 | 0.50 |
| 4 | 0.35 | 4 | 0.6 | 0.05 | 8 | 0.66 | 0.75 |
| 5 | 0.35 | 12 | 0.4 | 0.25 | 11 | 0.82 | 0.75 |
| 6 | 0.45 | 10 | 0.4 | 0.65 | 11 | 0.10 | 0.25 |
| 7 | 0.45 | 6 | 0.7 | 0.65 | 8 | 0.58 | 0.50 |

Apart from these differences, each model was run with the following similar properties: objective = binary: logistic; booster = gbtree. An up-to-date description of the exact role of each parameter and its possible values is provided in <https://xgboost.readthedocs.io/en/latest/parameter.html>.
