# Supplementary material for: Improved multi-parametric prediction of tissue outcome in acute ischemic stroke patients using spatial features
Source: PLoS One. 2020 Jan 24;15(1):e0228113. doi: 10.1371/journal.pone.0228113 (PMC6980585; doi:10.1371/journal.pone.0228113)
Supplement: S2 Table — Best results according to each metric are marked in bold text. Significant differences to this best-performing method computed with a one-sided paired student’s t-test are marked with a star (*) for a confidence interval of 95% (p < 0.05) and two stars (**) for a confidence interval of 99% (p < 0.01). Nominal p-values are reported without correction for multiplicity, similarly as in [23]. ADC = apparent diffusion coefficient, PWI = perfusion-weighted MRI parameters, MNI = MNI coordinates, LP = lesion probability. (DOCX) [file pone.0228113.s002.docx]

**S2 Table. Average ROC AUC and Dice results from the leave-one-patient-out cross-validations for each model.**

| Model | Features | Setting | mean ROC AUC | mean Dice | training time (s) |
| --- | --- | --- | --- | --- | --- |
| LR | ADC + PWI |  | 0.813±0.107** | 0.317±0.220** | 41 |
| LR | ADC + PWI + MNI |  | 0.827±0.100** | 0.292±0.229** | 48 |
| LR | ADC + PWI + LP |  | 0.874±0.108** | 0.319±0.238** | 45 |
| LR | ADC + PWI + MNI + LP |  | 0.877±0.099** | 0.322±0.232** | 44 |
| RF | ADC + PWI |  | 0.826±0.104** | 0.341±0.218** | 614 |
| RF | ADC + PWI + MNI |  | 0.891±0.092 | 0.383±0.226** | 628 |
| RF | ADC + PWI + LP |  | 0.883±0.104** | 0.371±0.227** | 622 |
| RF | ADC + PWI + MNI + LP |  | 0.889±0.092 | 0.368±0.228** | 758 |
| XGB | ADC + PWI | 1 | 0.814±0.118** | 0.321±0.222** | 45 |
| XGB | ADC + PWI | 2 | 0.826±0.104** | 0.337±0.218** | 98 |
| XGB | ADC + PWI | 3 | 0.827±0.108** | 0.337±0.222** | 146 |
| XGB | ADC + PWI | 4 | 0.825±0.107** | 0.325±0.223** | 62 |
| XGB | ADC + PWI | 5 | 0.821±0.113** | 0.322±0.224** | 80 |
| XGB | ADC + PWI | 6 | 0.828±0.105** | 0.346±0.221** | 107 |
| XGB | ADC + PWI | 7 | 0.830±0.105** | 0.346±0.220** | 77 |
| XGB | ADC + PWI + MNI | 1 | 0.852±0.106** | 0.336±0.214** | 44 |
| XGB | ADC + PWI + MNI | 2 | 0.883±0.092** | 0.374±0.221** | 103 |
| XGB | ADC + PWI + MNI | 3 | **0.893±0.085** | 0.387±0.213 | 179 |
| XGB | ADC + PWI + MNI | 4 | 0.850±0.113** | 0.329±0.224** | 59 |
| XGB | ADC + PWI + MNI | 5 | 0.880±0.091** | 0.355±0.216** | 93 |
| XGB | ADC + PWI + MNI | 6 | 0.889±0.093* | 0.387±0.222 | 122 |
| XGB | ADC + PWI + MNI | 7 | 0.890±0.092 | 0.386±0.218 | 94 |
| XGB | ADC + PWI + LP | 1 | 0.867±0.100** | 0.353±0.224** | 55 |
| XGB | ADC + PWI + LP | 2 | 0.884±0.105* | 0.379±0.228** | 121 |
| XGB | ADC + PWI + LP | 3 | 0.890±0.093 | 0.384±0.225* | 175 |
| XGB | ADC + PWI + LP | 4 | 0.878±0.102** | 0.367±0.220** | 78 |
| XGB | ADC + PWI + LP | 5 | 0.875±0.102** | 0.365±0.222** | 93 |
| XGB | ADC + PWI + LP | 6 | 0.886±0.101* | 0.387±0.224* | 122 |
| XGB | ADC + PWI + LP | 7 | 0.888±0.101 | **0.395±0.229** | 95 |
| XGB | ADC + PWI + MNI + LP | 1 | 0.869±0.094** | 0.336±0.225** | 55 |
| XGB | ADC + PWI + MNI + LP | 2 | 0.881±0.097** | 0.374±0.220** | 144 |
| XGB | ADC + PWI + MNI + LP | 3 | 0.888±0.104 | 0.379±0.226** | 202 |
| XGB | ADC + PWI + MNI + LP | 4 | 0.876±0.094** | 0.351±0.226** | 80 |
| XGB | ADC + PWI + MNI + LP | 5 | 0.887±0.097* | 0.375±0.231** | 120 |
| XGB | ADC + PWI + MNI + LP | 6 | 0.887±0.098* | 0.386±0.224 | 157 |
| XGB | ADC + PWI + MNI + LP | 7 | 0.890±0.097 | 0.381±0.228** | 114 |

Best results according to each metric are marked in bold text. Significant differences to this best-performing method computed with a one-sided paired student’s t-test are marked with a star (*) for a confidence interval of 95% (p < 0.05) and two stars (**) for a confidence interval of 99% (p < 0.01). Nominal p-values are reported without correction for multiplicity, similarly as in [22]. ADC = apparent diffusion coefficient, PWI = Perfusion-weighted MRI parameters, MNI = MNI coordinates, LP = lesion probability.
