# Supplementary material for: Improved multi-parametric prediction of tissue outcome in acute ischemic stroke patients using spatial features
Source: PLoS One. 2020 Jan 24;15(1):e0228113. doi: 10.1371/journal.pone.0228113 (PMC6980585; doi:10.1371/journal.pone.0228113)
Supplement: S1 Fig — Shapley value decomposition maps of the predictions from the best XGB models incorporating DWI and PWI (setting 7; rows 1–2), DWI, PWI, and MNI coordinates (setting 3; rows 3–4), DWI, PWI and LP (setting 7; rows 5–6). (DOCX) [file pone.0228113.s003.docx]

**S1 Fig. Shapley value decomposition maps.** Shapley value decomposition maps of the predictions from the best XGB models incorporating DWI and PWI (setting 7; rows 1-2), DWI, PWI, and MNI coordinates (setting 3; rows 3-4), DWI, PWI and LP (setting 7; rows 5-6).

[S1 Fig]
