# Supplementary figures and images for: Correction: Improved multi-parametric prediction of tissue outcome in acute ischemic stroke patients using spatial features
Source: PLoS One. 2020 Mar 12;15(3):e0230653. doi: 10.1371/journal.pone.0230653 (PMC7067434; doi:10.1371/journal.pone.0230653)

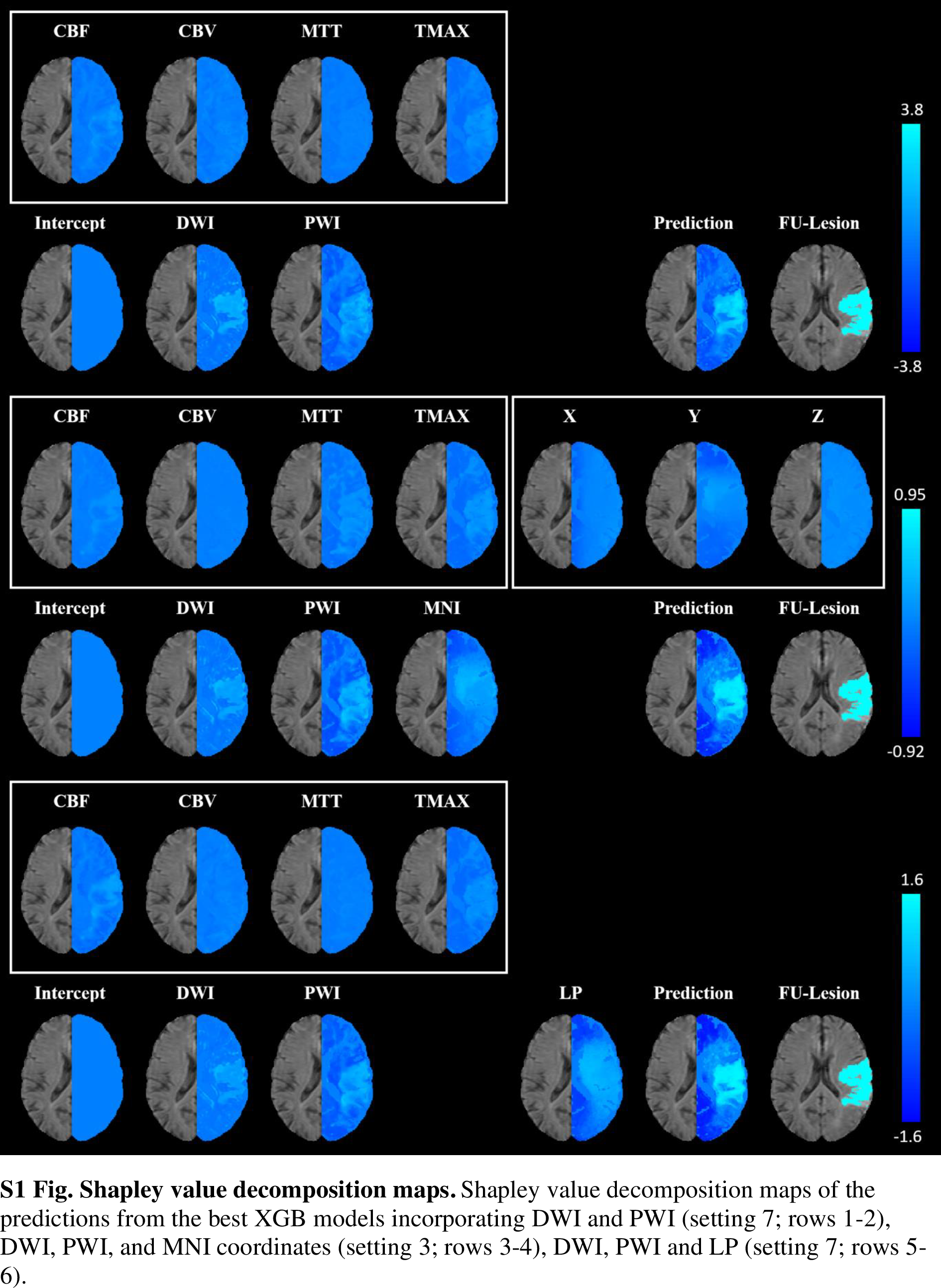

Supplement: S1 Fig — Shapley value decomposition maps of the predictions from the best XGB models incorporating DWI and PWI (setting 7; rows 1–2), DWI, PWI, and MNI coordinates (setting 3; rows 3–4), DWI, PWI and LP (setting 7; rows 5–6). (TIF) [file pone.0230653.s001.tif]
